# Sex-specific remodeling of proteasome complexes in lymph nodes of aged BTBR mice

**DOI:** 10.3389/fragi.2026.1864375

**Published:** 2026-07-06

**Authors:** Francesca Monittola, Michela Bruschi, Sofia Masini, Domenico Pio Losito, Mauro Magnani, Luigia Rossi, Alessandra Fraternale, Rita Crinelli

**Affiliations:** Department of Biomolecular Sciences, Section of Biochemistry and Biotechnology, University of Urbino Carlo Bo, Urbino, Italy

**Keywords:** aging, immunoproteasome, lymph nodes, PA28, sex

## Abstract

Lymph node architecture and function are markedly altered with aging. We previously demonstrated that the lymph nodes of old C57BL/6 and BTBR male mice contain reduced levels of PA28αβ-capped proteasomes compared to young mice. Here, we examined aged BTBR females relative to aged males and found that proteasome remodeling occurs in a sex-specific manner. Specifically, old female exhibited higher immunoproteasome activity than old male, despite similar levels of proteasome and immunoproteasome subunit expression and proteasome complex assembly. In female mice activity was mostly driven by PA28αβ-capped 20S proteasomes that declined with age in males. These results highlight intriguing differences in proteasome assembly and activity between males and females providing evidence for sex-dependent changes in immunosenescence.

## Introduction

Advancing age leads to immunosenescence, a progressive decline in immune function that increases susceptibility to infections, cancer, and autoimmune diseases, and reduces vaccine responsiveness. Lymph nodes are secondary lymphoid organs that play a pivotal role in initiating adaptive immunity, providing both the structural framework and the specialized microenvironment necessary to support immune-cell activation and interaction. A substantial body of evidence indicates that lymph node architecture and function are markedly altered with aging, and this deterioration contributes significantly to overall immune system dysfunction (Ng and Tay, 2025). Age-related lymph node remodeling is characterized by fibrosis and lipomatosis, diminished immune-cell recruitment and spatial organization, and a reduced number and size of germinal centers, all of which ultimately impair effective responses to pathogens and antigens. Moreover, recent studies indicate that, beyond shifts in the composition and activity of immune-cell subpopulations, most cells in the lymph nodes of aged mice display multiple senescence markers, implying that immune cells themselves undergo senescence (Deng et al., 2025).

The loss of proteostasis is widely regarded as a major driver of senescence, but its contribution to immune-cell dysfunction remains unclear. Addressing this gap requires a better understanding of the mechanisms that regulate proteostasis in immune cells and how these mechanisms change in senescent cells (Gressler et al., 2023). Proteasome dysfunction is a major contributor to altered proteostasis, however the dynamics of specific proteasome complex subtypes, particularly within immune cells and tissues, are largely unexplored (Raynes et al., 2016). The proteasome is a proteolytic complex composed of a 20S core particle, which consists of four rings arranged in αββα configuration. Three of the β-type subunits (β1, β2, and β5) exhibit distinct proteolytic activities: caspase-like, trypsin-like, and chymotrypsin-like, respectively. Immunoproteasome is a specialized variant of the standard proteasome in which the three β-catalytic subunits are replaced by their counterparts β1i, β2i and β5i, forming the i20S core particle (Murata et al., 2018). Both the 20S and i20S proteasome activities are regulated by association with regulatory particles (RPs), such as PA28αβ and 19S—the latter of which enables ATP- and ubiquitin-dependent degradation of protein substrates. Immunoproteasome subunits are predominantly expressed in immune cell subsets where they are thought to enhance and broaden MHC class I antigen presentation, promote the development of T cell subsets, and modulate cytokine production, thereby regulating both adaptive and innate immune responses (Murata et al., 2018). Besides its role in immunity, the immunoproteasome is also involved in maintaining protein homeostasis under stress and pro-inflammatory conditions (Basler and Groettrup, 2021).

Sex is considered a biological variable, impacting different aspects of health and illness, including aging. We previously demonstrated that the lymph nodes of aged C57BL/6 and BTBR male mice as well contain lower levels of PA28αβ-capped proteasomes compared to those of young mice (Monittola et al., 2025). Owing to the availability of lymph nodes from BTBR animals of both sexes, in this work, we explored whether age-associated proteasome dynamics observed in lymph nodes of males also occur in females. Indeed, aging affects the immune system differently in females and males, and sex-specific differences in immune responses have also been observed in species other than humans (Baker et al., 2025). Indeed, Jenkins et al. recently showed that the age-related decline in proteasome activity is neither uniform across tissues nor consistent between sexes, and that variability tends to increase with age, suggesting that aging may introduce additional layers of heterogeneity (Jenkins et al., 2020).

## Methods

### Animals

Male and female BTBR mice were obtained from an in-house colony bred in the animal facility of the Department of Biomolecular Sciences, University of Urbino Carlo Bo. Two groups of animals were used: the first group comprised five young and five old males and two young and eight aged females raised with the male cohort analyzed in previous studies (Monittola et al., 2025). A second group consisting of three young and four aged female mice was included to increase the number of young females. The animals were housed in standard cages (3–5 animals per cage) with a light-dark cycle of 12 h and under controlled conditions of temperature (22 °C ± 1 °C), humidity (60%), and air change (every 12 h). All mice were fed with a Teklad global 18% protein rodent diet (Teklad, Harlan Laboratories Inc., Madison, WI) and water *ad libitum*. Inguinal, axillary, and submandibular lymph nodes were obtained from young male (3 month-old), aged male (17.8 ± 1.3 month-old), young female (3.8 ± 1 month-old) and aged female (17.9 ± 1.5 month-old) mice. Three-to six-month-old mice are classified as young, whereas eighteen-month-old mice are aged and are often preferred over 20–24-month-old mice because they exhibit a lower incidence of severe age-related pathologies and reduced mortality, while still displaying a clear aging phenotype. Organs were excised, immediately frozen in liquid nitrogen, and stored at −80 °C (authorization number 486/2017-PR Italian Ministry of Health). Tissue freezing was necessary to collect samples and analyze them in parallel. All experiments were conducted in accordance with European legislation (2010/63/EU) and Italian national legislation (DL26/2014) regulating the use of animals for research and the guidelines of Istituto Superiore di Sanità on the use and care of laboratory animals. The number of mice was established as the minimum number to obtain statistically significant differences.

### Lymph node extracts

Lymph node extracts were prepared from frozen tissues by homogenization with a Teflon pestle in non-denaturing lysis buffer containing 10 mM Tris–HCl (pH 7.5), 5 mM MgCl_2_, 10 mM NaCl, and 10% (v/v) glycerol, supplemented with 1 mM DTT, 2 mM ATP, and a protease inhibitor cocktail. Although freezing can affect protein integrity, several protocols for proteasome analysis use frozen samples as starting material or involve freeze–thaw cycles during extract preparation (Ruiz-Romero et al., 2025; Yazgili et al., 2021). After centrifugation at 20,000 × g for 20 min at 4 °C, the supernatant was collected and transferred to a new tube. Protein concentration was determined using the Bradford assay with bovine serum albumin as the standard. Aliquots were stored at −80 °C or diluted in SDS sample buffer for western blot analysis.

### Proteasome activity

Proteasome activities were assayed in lymph node extracts using fluorogenic synthetic substrates specific for trypsin-like (Boc-LLR-AMC), chymotrypsin-like (Suc-LLVY-AMC), caspase-like (Z-LLE-AMC), β1i (Ac-PAL-AMC), and β5i (Ac-ANW-AMC) activities. The release of AMC following peptide hydrolysis was monitored using a FLUOstar Optima fluorimeter (BMG Labtech) set to excitation/emission wavelengths of 355/460 nm. The incubation mixture contained 50 mM HEPES/KOH (pH 7.8), 5 mM KCl, 2.5 mM ATP, and 25 mM MgCl_2_. Protein and substrate concentrations were selected based on pilot experiments to ensure measurements within the linear range of the response. Fluorometric units (FU) were plotted as a function of time and proteasome activity was calculated from the slope after linear regression analysis.

### Western immunoblotting

Proteins were resolved by SDS–PAGE and transferred onto PVDF membranes. After transfer, total protein was detected by incubating the membranes with the No-Stain reagent (Invitrogen) according to the manufacturer’s instructions. Images were acquired using a ChemiDoc MP imaging system (Bio-Rad), and total protein content in each lane was quantified using Image Lab software version 5.2.1 (Bio-Rad). After blocking, membranes were incubated with the following primary antibodies: anti-α1,2,3,5,6,7 (#PW8195, pan α; Enzo); anti-PSMB6/β1 (#A4053), anti-PSMB7/β2 (#14771), anti-PSMB10/β2i (#A5452), anti-19S PSMC6/Rpt4 (#A5377), and anti-PSME2/PA28β (#A5562) (ABclonal); anti-PSMB8/β5i (#13635; Cell Signaling Technology); anti-PSMB9/β1i (C-terminus) (#AP21207b) and anti-PSMB5/β5 (#ALS17241) (Abcepta). HRP-conjugated secondary antibodies were obtained from Bio-Rad. Signals were detected using the WesternBright ECL kit (Enhanced Chemiluminescence; Advansta), and immunoreactive bands were visualized using the ChemiDoc MP system. Band intensities were quantified with Image Lab software and normalized to total protein content as determined by the No-Stain reagent.

### Native PAGE and in-gel activity

Lymph node extracts (20 µg) were separated on 4% native PAGE gels using a running buffer containing 90 mM Tris, 90 mM borate, 5 mM MgCl_2_, 1 mM EDTA, and 0.5 mM ATP for 2.5 h at 150 V and 4 °C. For in-gel activity assays, gels were incubated at 37 °C in the dark with Boc-LRR-AMC to assess trypsin-like activity, sLLVY-AMC for chymotrypsin-like activity and with Ac-PAL-AMC and Ac-ANW-AMC to assess β1i and β5i activity, respectively, and subsequently imaged under UV light using a ChemiDoc MP imaging system (Bio-Rad) (Monittola et al., 2023). Gels were then denatured in solubilization buffer (2% (w/v) SDS, 66 mM Na_2_CO_3_, and 1.5% (v/v) β-mercaptoethanol). Proteins were transferred onto PVDF membranes and probed with antibodies against α subunits of the core particle, PA28β of the PA28α/β regulator and β5i of the immunoproteasome. Image acquisition and band quantification were performed as described above.

### Statistical analysis

Statistical analysis of the data was performed by GraphPad Prism version 8.4.2 for Windows. Group differences were analyzed using the Kruskal–Wallis test followed by Dunn’s multiple comparisons test (denoted by asterisk). Differences between two animal groups were assessed using the Mann–Whitney test (denoted by §). Values were considered significant at p values ≤ 0.05.

## Results

**FIGURE 1:**
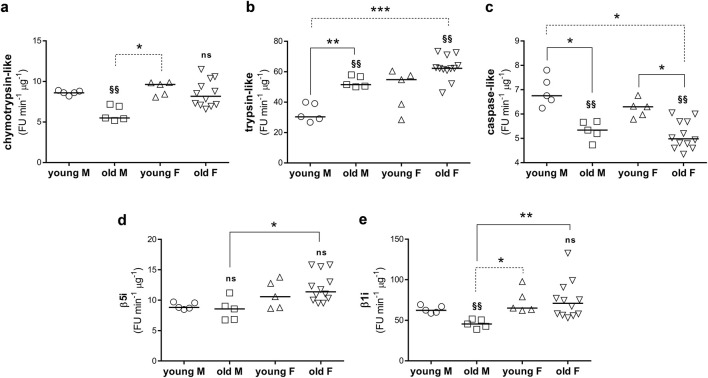
Proteasome activity in lymph node extracts. Proteasome activity was measured in lymph node extracts using fluorogenic substrates specific for distinct proteasome **(a–c)** and immunoproteasome **(d,e)** activities. The histograms represent the mean ± S.D. of the fluorometric unit (FU)/minute/μg of total proteins. Significance thresholds are indicated as p < 0.001 (***/§§§), p < 0.01 (**/§§), and p < 0.05 (*). *Significance of multiple comparisons; § and ns (not significant) significance vs. the relative young group.

Proteasome activity was preliminarily measured in free extracts using various fluorogenic peptides specific to the chymotrypsin-like, trypsin-like and caspase-like activities, as well as for the β5i- and β1i-associated activities of the immunoproteasome (Figure 1). The results indicate that constitutive proteasome activities were similarly affected by aging in the lymph nodes of both male and female mice, with an increase in trypsin-like activity (Figure 1b) and a decrease in caspase-like activity (Figure 1c). Increased trypsin-like activity in females compared to males has been reported in other tissues such as spleen, kidney and intestine (Jenkins et al., 2020). In contrast, chymotrypsin-like activity and β1i activity were reduced exclusively in males (Figures 1a,e). Multiple comparison analyses further revealed that the most pronounced differences occurred between aged groups, with aged males exhibiting significantly lower immunoproteasome activities relative to aged females.

Based on these findings, we next investigated whether the observed differences could be attributed to differential expression of proteasome/immunoproteasome and/or regulatory particle subunits, using SDS-PAGE followed by Western immunoblotting. Quantification of the immunoreactive bands, normalized to total protein content, revealed no significant differences between groups, although β1, β5, and β5i subunits tended to be less expressed in females (Supplementary Figure S1).

**FIGURE 2:**
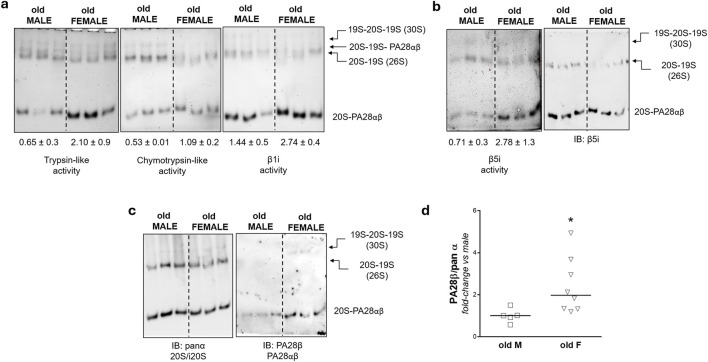
Native-PAGE analysis of proteasome complexes. Lymph node homogenates were separated on a 4% (w/v) polyacrylamide gel run under native conditions. In-gel activity was detected under UV light using a ChemiDoc MP imaging system and quantified with the Image Lab software **(a,b)**. Numbers below the panels indicate the ratio between 20S-PA28αβ and 26S+30S signals. Proteins were subsequently subjected to Western blot analysis with antibodies specific for β5i, PA28β, and α subunits (panα; anti-α1,2,3,5,6,7) **(b,c)**. The graph shows the quantification of 20S-PA28αβ complexes normalized to 20S content, as determined by the ratio of PA28β to pan-α antibody signals **(d)**. Quantifications were performed within the linear range of the antibody signals. Data from old female mice are expressed as fold change relative to the mean value of old male mice ± S.D. p < 0.05 (*). IB: immunoblot.

The levels of functional proteasome particles, however, depend not only on subunit expression, but also on their quantitative and qualitative assembly into proteolytic complexes, as well as on the association of the 20S core with RPs. To determine the activity and composition of proteasome subtypes in lymph node extracts, we performed in-gel proteasome activity assays followed by immunoblotting with antibodies against core and regulatory subunit complexes. Compared to males, most of the proteasome activities in females were strongly associated with PA28αβ-capped core particles (20S) (Figures 2a,b), despite the amount and composition of 20S particles did not change as demonstrated by β5i and pan-α staining of native gels (Figures 2b,c). Quantification of the ratio between 20S-PA28αβ- and 26S+30S-associated activities, shown below each panel, further supports the conclusion that females had higher 20S-PA28αβ-driven activity (Figures 2a,b). Accordingly, females contained more PA28αβ-capped 20S proteasomes than males, as determined by increased PA28β staining of complexes co-migrating with the 20S core (Figure 2c). Quantification of the PA28β signal, normalized to the pan-α signal associated with the 20S core particle, showed that females had almost 2.5 times more PA28αβ-capped proteasomes than males accounting for increased immunoproteasome activity despite similar levels of immunosubunit expression (Figure 2d). Indeed, PA28αβ capping can enhance fluorogenic peptide degradation rates; this effect has been attributed to PA28αβ’s ability to induce 20S gate-opening (Cascio, 2025). Differences in proteasome activity, independently of proteasome component levels, have also been reported by others, highlighting that changes in proteasome dynamics cannot be fully captured by conventional proteomic approaches and underscoring the need to distinguish between 20S and 26S proteasomes (Jenkins et al., 2020).

## Discussion

Notwithstanding the progress made in highlighting sex-associated dimorphisms in immune responses, the molecular mechanisms underlying these differences remain poorly understood. Single-cell transcriptomic analyses have demonstrated that aging exerts a greater impact on the immune system than sex, while simultaneously amplifying sex-related differences (Huang et al., 2021).

Examination of differentially expressed genes revealed that circulating immune cells from aged males exhibit increased oxidative stress and a heightened inflammatory state compared to cells from aged females (Huang et al., 2021). Under oxidative stress conditions, PA28αβ, in conjunction with the i20S core particle, is strongly induced to remove oxidatively damaged proteins, thereby contributing with the 20S particle to the maintenance of proteostasis, as demonstrated by PA28αβ knockout studies (Pickering et al., 2012). It has been demonstrated that during aging, it is the activity of the 20S proteasome that declines rather than that of the 26S proteasome, thereby impairing cells’ ability to degrade oxidized proteins and contributing to the overall loss of protein homeostasis (Raynes et al., 2016). Consequently, it could be speculated that in females a higher content of PA28αβ-capped immunoproteasomes, may contribute to better control of oxidative stress and inflammation associated with aging (inflammaging).

Preservation of PA28αβ-capped immunoproteasomes in aged females may also contribute to sex-specific differences in immune function and susceptibility to various pathologies during aging. Indeed, females typically mount more robust innate and adaptive immune responses, enabling more efficient pathogen clearance and stronger responses to vaccination compared to males. However, sex-related differences in the immune response appear to be dependent on multiple factors such as the pathogen, antigen, vaccine platform, as well as host genetic, epigenetic, and microbiota-related factors (Klein et al., 2015; Wilkinson et al., 2022). As a trade-off, females are at higher risk of developing autoimmune diseases but are generally less susceptible to malignancies (Forsyth et al., 2024). Importantly, PA28αβ does not act on specific antigens *per se*, but rather modulates proteasomal cleavage specificity and peptide release, thereby editing the repertoire of peptides generated for MHC-I presentation (Sijts et al., 2002). Its effects are therefore epitope- and context-dependent rather than antigen-specific. In this context, preservation of PA28αβ-capped immunoproteasomes in aged females may preferentially sustain the generation and presentation of subsets of intracellular viral and tumor-derived epitopes whose production is particularly sensitive to proteasomal processing. This could contribute to the maintenance of CD8^+^ T-cell-mediated immunity and immune surveillance during aging.

The role of the PA28αβ/immunoproteasome complex in broadening the repertoire of MHC-I antigens and in promoting the degradation of unfolded or oxidized proteins appears to be substrate-specific and context-dependent, making it challenging to assign a specific function to this proteolytic particle in immune regulation (Cascio, 2025). Consequently, whether PA28αβ-capped i20S proteasomes influence the survival, proliferation, or retention of immune cells in lymph nodes remains to be determined. Intriguingly, peptides generated by the proteasome have emerged as regulators of several intracellular pathways, including antimicrobial immunity, new protein synthesis, and metabolism, raising the possibility that the PA28αβ regulator may elicit immune signals beyond than antigenic peptides (Brennan et al., 2025).

In this study, we focused on the PA28αβ-capped 20S immunoproteasome fraction, in which the most pronounced sex- and age-associated differences were observed. Consequently, the role of 26S proteasome activity was not specifically addressed, although it contributes to both immune regulation and general proteostasis and warrants further investigation.

Finally, BTBR mice show an activated immune system with a predominant Th2 profile compared to other mice strains commonly used to study ageing. The development of proinflammatory and autoimmune-prone immunologic characteristics of BTBR mice was not influenced by sex-linked genetic differences (Heo et al., 2011). Considering that alterations in inflammatory and immune pathways are key drivers of the aging process, the BTBR model may represent a valuable tool for investigating the impact of immune dysregulation during aging in comparative studies involving C57BL/6 mice (Li et al., 2023). Our previous comparative analysis between BTBR and C57BL/6 male mice demonstrated that PA28αβ proteasome capping display comparable age-related changes across strains (Monittola et al., 2025). Furthermore, BTBR mice share a common MHC H-2^b^ haplotype with C57BL/6 mice (Schwartzer et al., 2017). Nevertheless, we cannot exclude the possibility that other parameters may be differentially affected during aging, and further studies will be required to address this aspect.

Overall, these observations underscore the need to extend aging studies to both sexes, which may help better elucidate the underlying mechanisms and develop sex-specific therapeutic interventions.

## Data Availability

The original contributions presented in the study are included in the article/Supplementary Material, further inquiries can be directed to the corresponding authors. Data Sheet 1 contains uncropped images, including samples not shown in the main figures that were used only for quantification purposes. Data Sheet 2 contains Supplementary Figure S1.
