# Supplementary material for: Sex-specific remodeling of proteasome complexes in lymph nodes of aged BTBR mice
Source: Front Aging. 2026 Jul 6;7:1864375. doi: 10.3389/fragi.2026.1864375 (PMC13381488; doi:10.3389/fragi.2026.1864375)
Supplement: Supplementary file 1 [file DataSheet2.pdf]

SUPPLEMENTARY FIGURE

Sex-specific remodeling of proteasome complexes in lymph nodes of aged BTBR mice

Francesca Monittola*, Michela Bruschi, Sofia Masini, Domenico Pio Losito, Mauro Magnani, Luigia Rossi, Alessandra Fraternale, Rita Crinelli*

Department of Biomolecular Sciences, Section of Biochemistry and Biotechnology, University of Urbino Carlo Bo, Urbino (PU), Italy

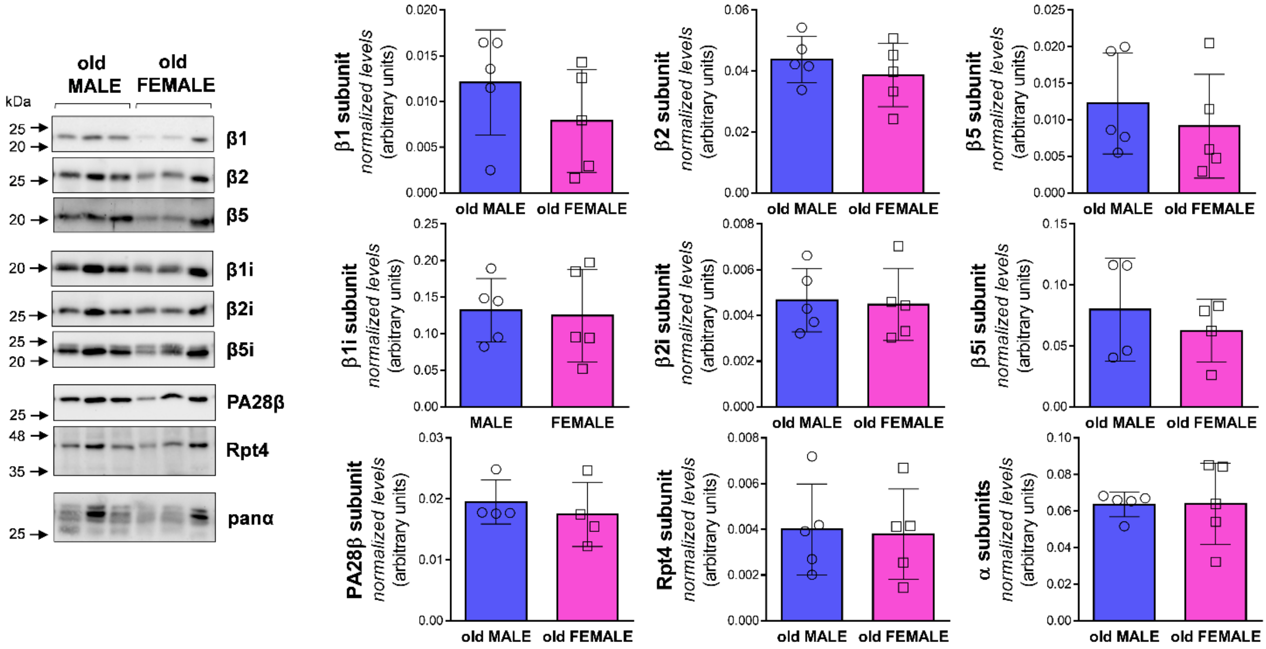

Figure S1. Proteasome, immunoproteasome and regulatory particle subunit expression levels. Native lysates were denatured in sample buffer, separated by SDS polyacrylamide gel electrophoresis (SDS-PAGE), transferred onto PVDF membrane, and probed with specific antibodies against α-subunits (panα: anti-α1,2,3,5,6,7), catalytic subunits β1, β2, β5 of the 20S complex and β1i, β2i, β5i of i20S, and RP subunits PA28β and Rpt4 of PA28αβ and 19S regulators, respectively. Immunoreactive bands were detected using a Chemidoc system and quantified with Image Lab software. Protein levels were normalized on total protein content detected using the NoStain Reagent. Histograms represent the mean ± S.D of arbitrary units. Images show three representative samples.
