# Supplementary material for: Sex-specific remodeling of proteasome complexes in lymph nodes of aged BTBR mice
Source: Front Aging. 2026 Jul 6;7:1864375. doi: 10.3389/fragi.2026.1864375 (PMC13381488; doi:10.3389/fragi.2026.1864375)
Supplement: Supplementary file 2 [file DataSheet1.PDF]

Figure 2. Uncropped images (red square) and samples not shown used for quantitation (black square)

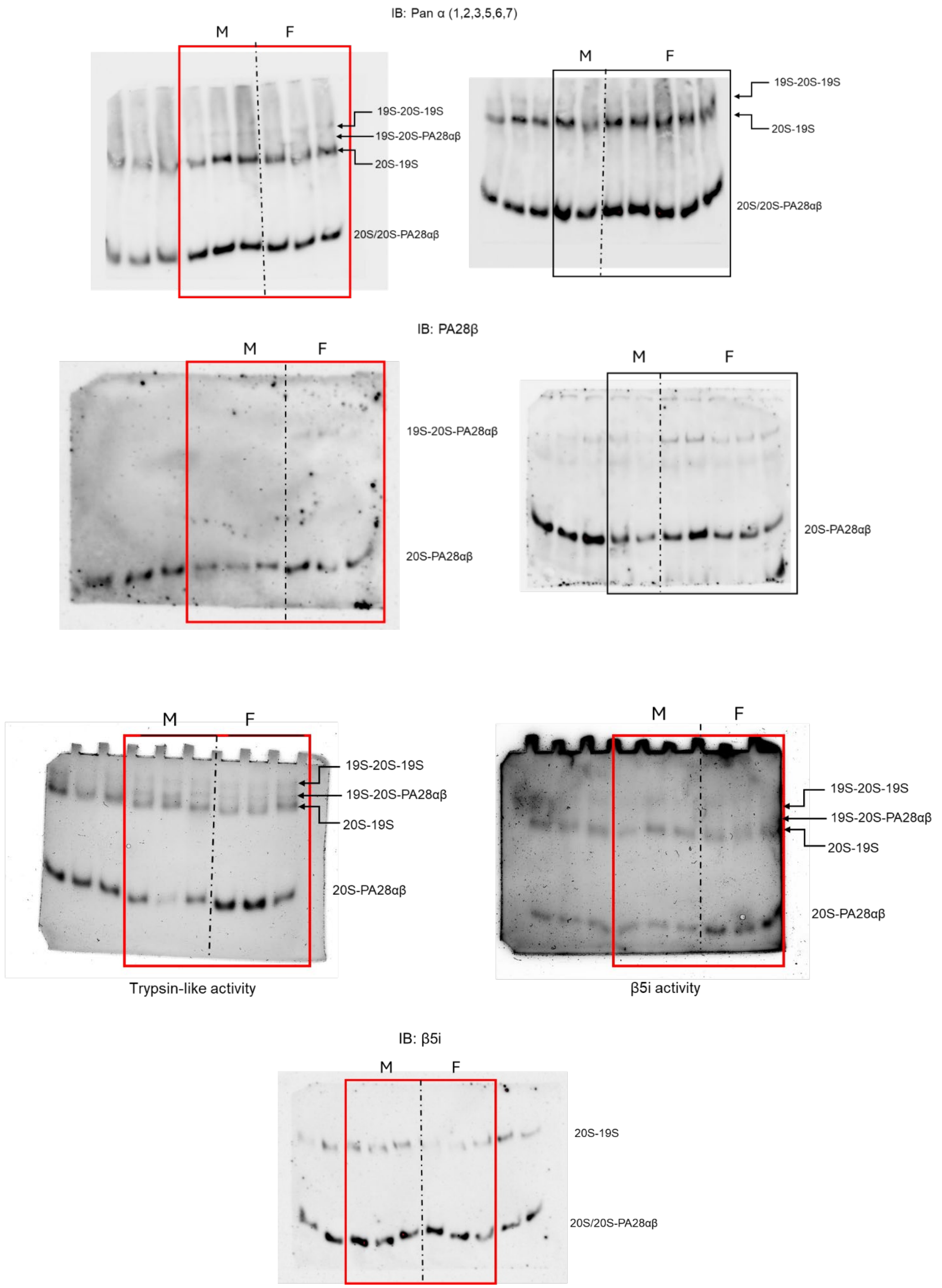

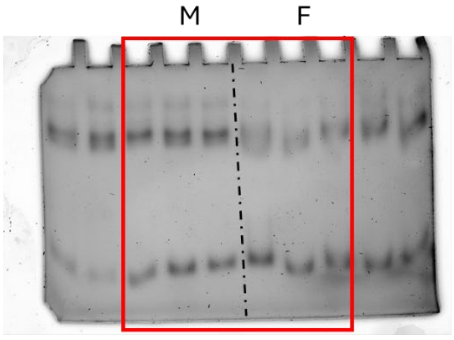

Chymotrypsin activity

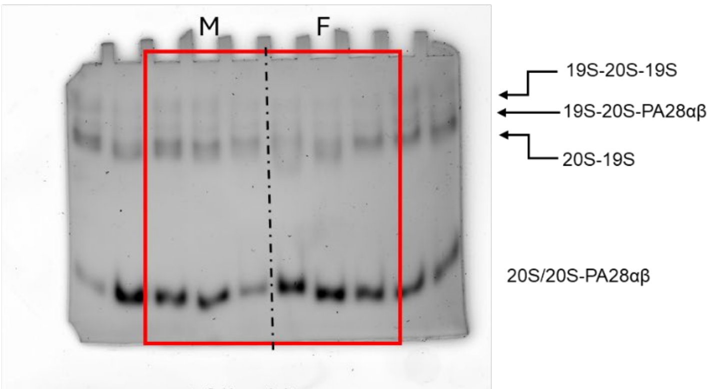

β1i activity

Figure S1. Uncropped images (red circle) and samples not shown used for quantitation (black circle)

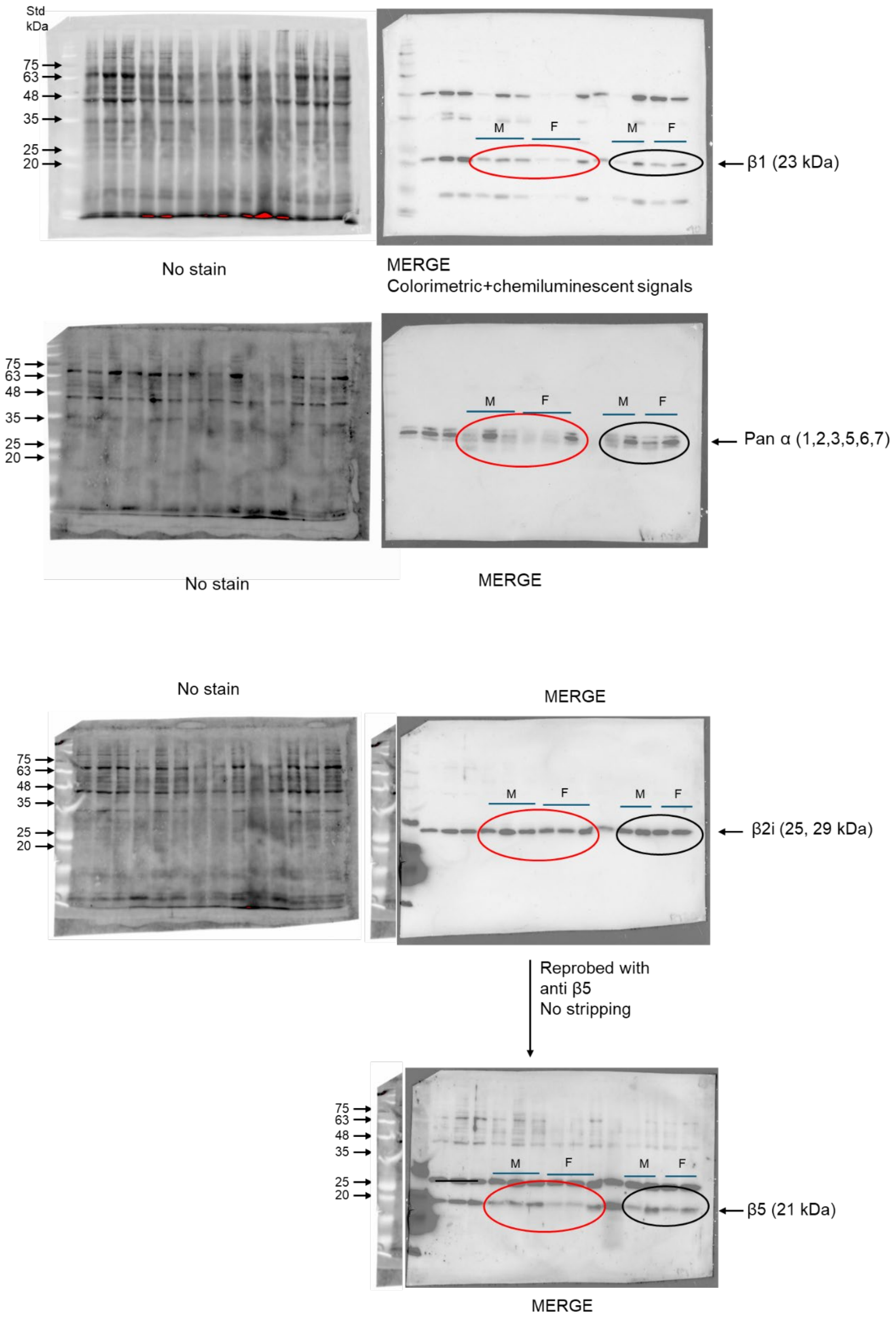

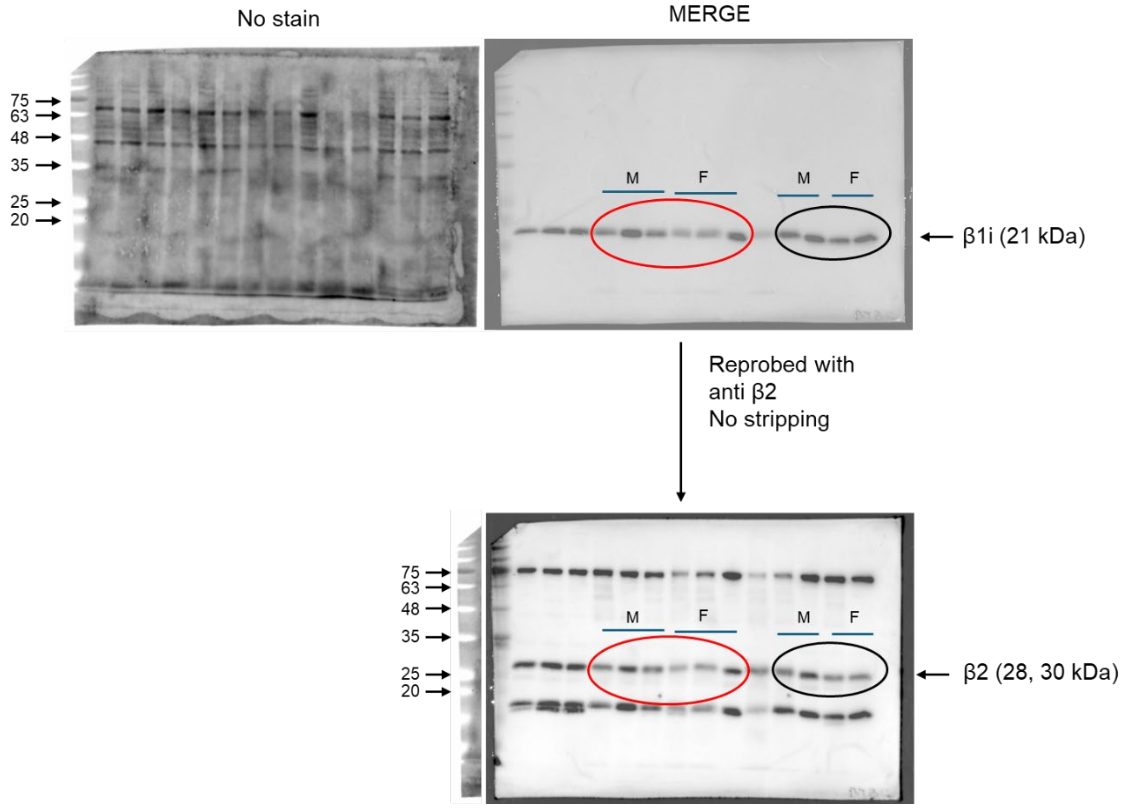
No stain
MERGE
75
63
48
35
25
20
M
F
M
F
β1i (21 kDa)
Reprobed with
anti β2
No stripping
75
63
48
35
25
20
M
F
M
F
β2 (28, 30 kDa)

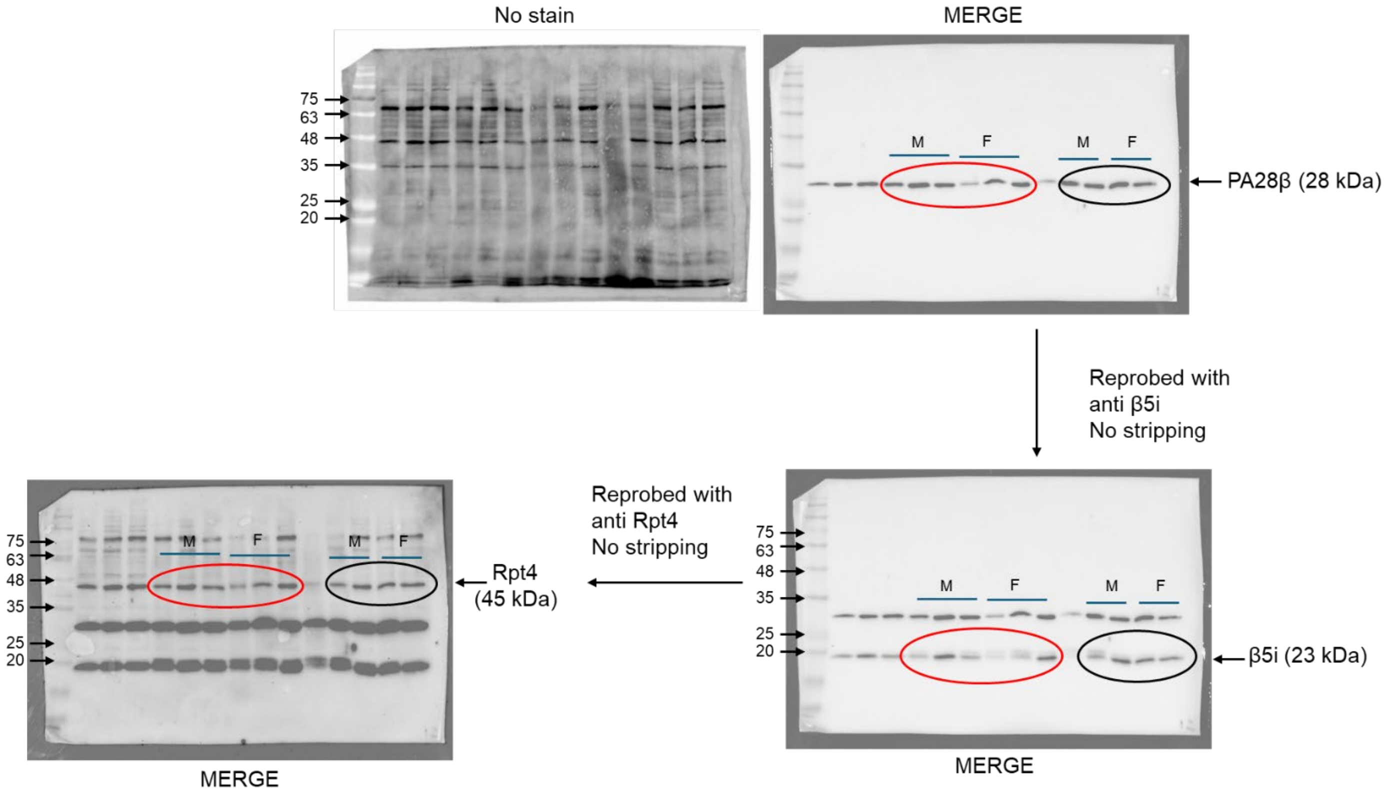
No stain
MERGE
75
63
48
35
25
20
M
F
M
F
PA28β (28 kDa)
Reprobed with
anti β5i
No stripping
75
63
48
35
25
20
M
F
M
F
β5i (23 kDa)
MERGE
Reprobed with
anti Rpt4
No stripping
75
63
48
35
25
20
M
F
M
F
Rpt4
(45 kDa)
MERGE
